# Quantitative imagery analysis of spot patterns for the three-haplogroup classification of* Triatoma dimidiata* (Latreille, 1811) (Hemiptera: Reduviidae), an important vector of Chagas disease

**DOI:** 10.1186/s13071-021-04598-5

**Published:** 2021-01-29

**Authors:** Daryl D. Cruz, Dennis Denis, Elizabeth Arellano, Carlos N. Ibarra-Cerdeña

**Affiliations:** 1 Centro de Investigación en Biodiversidad y Conservación (CIByC), Universidad Autónoma del Estado de Morelos (UAEM), Cuernavaca, Morelos México; 2 Departamento de Biología Animal y Humana, Facultad de Biología, Universidad de La Habana, Havana, Cuba; 3 Departamento de Ecología Humana, Centro de Investigación y de Estudios Avanzados del IPN (CINVESTAV), Unidad Mérida, Yucatán, México

**Keywords:** Cryptic species, Coloring pattern, Species complex, Taxonomy, Neural classification network

## Abstract

**Background:**

Spots and coloring patterns evaluated quantitatively can be used to discriminate and identify possible cryptic species. Species included in the *Triatoma dimidiata* (Reduviidae: Triatominae) complex are major disease vectors of Chagas disease. Phylogenetic studies have defined three haplogroups for Mexico and part of Central America. We report here our evaluation of the possibility of correctly discriminating these three *T. dimidiata* haplogroups using the pattern of the dorsal spots.

**Methods:**

Digital images of the dorsal region of individuals from the three haplogroups were used. Image processing was used to extract primary and secondary variables characterizing the dorsal spot pattern. Statistical analysis of the variables included descriptive statistics, non-parametric Kruskal–Wallis tests, discriminant function analysis (DFA) and a neural classification network.

**Results:**

A distinctive spot pattern was found for each haplogroup. The most differentiated pattern was presented by haplogroup 2, which was characterized by its notably larger central spots. Haplogroups 1 and 3 were more similar to each other, but there were consistent differences in the shape and orientation of the spots. Significant differences were found among haplogroups in almost all of the variables analyzed, with the largest differences seen for relative spot area, mean relative area of central spots, central spots Feret diameter and lateral spots Feret diameter and aspect ratio. Both the DFA and the neural network had correct discrimination values of > 90%.

**Conclusions:**

Based on the results of this analysis, we conclude that the spot pattern can be reliably used to discriminate among the three haplogroups of *T. dimidiata* in Mexico, and possibly among triatomine species. 
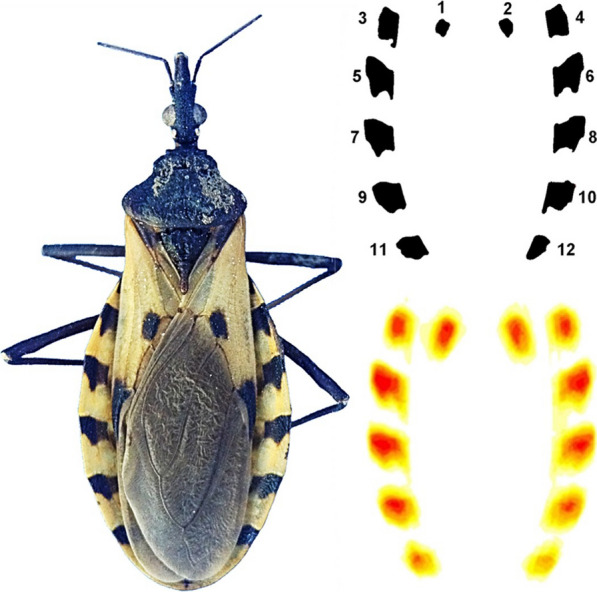

## Background

Genetic and morphological divergences associated with speciation processes may not appear at the same time or progress at the same rate [1]. The emergence of new species usually results from the isolation of populations due to geographic, ecological or behavioral barriers that can act individually or synergistically [2]. This can lead to populations that have substantial genetic differentiation that has not been expressed phenotypically (at least not obviously), giving rise to cryptic species [3]. Identifying cryptic species complexes is one of the most important challenges facing taxonomy in recent years [4].

The correct delimitation of cryptic species has important implications for research in many fields of biology, such as studies on biodiversity, conservation and behavioral ecology [4], and is frequently achieved using different types of data, such as molecular, ecological, behavioral and geometric morphometric data [5]. This combination of methods, known as integrative taxonomy [5, 6], is the surest and most precise way of determining species limits [7, 8].

Cryptic species in the genus *Triatoma* (main vectors of Chagas disease) have primarily been recognized using molecular tools [9–12], although both ecological and morphometric analyses have also been used [13]. Within the genus* Triatoma*, the *dimidiata* complex has received considerable attention, in part because it is one of the most widely distributed triatomine species complexes. It is the only triatomine bug that naturally occurs throughout the northern neotropical realm of North, Central and South America [14]. Analysis of genetic data has led to at least five new species being proposed as members of the *dimidiata* complex [15]. In addition, the species in this complex have different morphological patterns [16, 17]. In the field of epidemiological entomology, the delimitation of species of medical importance is vital for the establishment of efficient control strategies [13, 18]. Geometric morphometric techniques using landmarks [19–22] or body contour descriptors [23–26] have been used for this purpose, mainly because of the superiority of this approach over traditional morphometric methods [27] and because it is a much cheaper than, for example, molecular ones.

In Mexico, three haplogroups have been reported within the *T. dimidiata* complex. Haplogroup 1 (H1) is distributed east of the Isthmus of Tehuantepec and has been recently found in northern Guatemala. Haplogroup 2 (H2) is only found in Mexico and is distributed in two states along the Gulf of Mexico (Tabasco, Veracruz), five states in Central Mexico (Guanajuato, San Luis Potosí, Hidalgo, Puebla, Morelos), in small foci along the Pacific coast (Nayarit, Jalisco, Colima, Michoacán, Guerrero, Oaxaca) and, recently, in the Yucatán peninsula (Campeche, Yucatán) [9, 11]. Haplogroup 3 (h3) has only been recorded in Chiapas, Mexico [11].

Spot patterns are widely used to describe species in traditional taxonomy [28]. However, because spot pattern is highly variable due to its ecological functions, it is usually described in subjective, qualitative terms. Alternatively, using digital tools to quantify spot patterns can minimize bias, increase precision and allow automated identification processes [29]. However, since few studies use quantitative measurements of pattern properties for taxonomic purposes, evidence on the usefulness of color patterns to separate (or discriminate) species is still lacking [30].

The general body color of triatomines is black or spruce, with pattern elements ranging from light yellow to light brown, orange or red shades [31]. The lighter pattern elements can be present on any area of the body or appendages, and the color, intensity and distribution of these elements are of considerable importance for systematic purposes. The pattern of the connexivum region is particularly notable [32]. However, despite the taxonomic importance of these pattern elements, there have been very few quantitative studies of color and pattern variation in *Triatoma*. The first studies to quantify color patterns in a species of *Triatoma* were by carried out by Nattero et al. [33], who analyzed the melanic and non-melanic forms of domestic and peridomestic populations of *Triatoma infestans*, and by Carmona-Galindo et al. [34] who, in addition to other aspects, explored pattern variation as a function of elevation in triatomines from El Salvador, including populations of *T. dimidiata*. To date, the utility of the spot pattern to discriminate among haplogroups within the *dimidiata* complex or any other triatomine complex has not been explored.

Although there are no obvious external morphological differences based on our observations of *dimidiata* complex specimens, we hypothesize that the evaluation of more detailed quantitative differences in the spot pattern among haplogroups could be used to distinguish them morphologically. This method could potentially improve the separation criteria for known species and cryptic species in this group without the need for genetic data. This is possible because several species of triatomines have distinctive spot patterns, and these may be of relevant taxonomic value. In the study reported here, we evaluated the reliability of discriminating among the three *T. dimidiata* haplogroups reported for Mexico and part of Central América using the dorsal spot pattern. If successful, this technique could be extended to other species in this (and other) group and lay the foundations for an automated identification system to facilitate correct species recognition within the genus *Triatoma*. These systems could be employed by taxonomists, vector ecologists, health personnel, among others, interested in the rapid identification of these triatomines.

## Methods

### Sample information

Images of individuals from each of the haplogroups of *Triatoma dimidiata* were obtained from Gurgel-Gonçalves et al. [20]. These images are part of a collection of images of 51 triatomine species from Mexico and Brazil available for public use in the Dryad repository (http://dx.doi.org/10.5061/dryad.br14k). The original series of images that represent the species distributed in Mexico was taken from the following entomological collections in Mexico: Regional Center for Health Research, National Institute of Public Health of Mexico, Guanajuato State Public Health Laboratory, Benito Juárez Autonomous University of Oaxaca and the Autonomous University of Nuevo León, Monterrey, and details of how these images were taken are described in the referenced publications. We obtained 44, 30, and 40 images of individuals belonging to haplogroups 1, 2, and, 3 respectively; the haplogroup assignment of these individuals was corroborated genetically, and this corroboration constitutes a major factor for using these images in a quantitative analysis like our work [see 11]. In addition, the specimens in the photographs in [20] belong to the locations mentioned by Pech-May et al. [11], so the ecological variability of their distribution is included. From the 114 images, we selected only high-quality images that clearly captured the spot pattern, eliminating those cases where the spots were fused or covered by hyperchromatic wings (Additional file 1: Figure S1), resulting in a final sample of 101 images (39 for H1, 23 for H2, and 39 for H3).

### Image processing

**Fig. 1.**
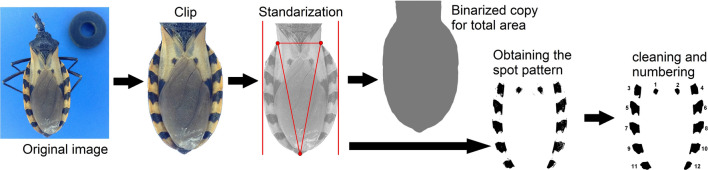
Steps in the image processing of three haplogroups of *Triatoma dimidiata* (Hemiptera: Reduviidae) for the analysis of spot patterns. Standardization includes isometric rescaling, translation and rotation following common reference guidelines. Spot removal and cleaning were done with a macro code in ImageJ. Copyright: Creative Commons Attribution 1.0 Universal (CC0 1.0) Public Domain Dedication license 163 (https://creativecommons.org/licenses/by/1.0). Images were modified from Gurgel-Gonçalves et al. [20], with permission

The images were processed to facilitate the extraction of standardized measurements of the spot pattern (Fig. 1). The abdomens were clipped manually, removing the legs and cutting off the head at the thorax level. The images were then aligned and re-scaled, using the insertion angles of the abdomen and thorax and the back of the body as references for alignment and scaling all individuals to the width of the first individual that was taken as a reference (image H10355). These transformations may slightly alter the shape and absolute values of the spot measurements, but they are essential for standardizing the spatial patterns of the spots and making them comparable, eliminating differences due to body shape or size, whose identifying value has been tested in previous studies [20, 26]. For this reason, the quantitative estimates of areas in this study are always expressed relative to the total area of the abdomen and linear measurements as relative to the square root of the total abdomen area.

Processing for spot pattern extraction included removing color information (desaturation) and reducing levels to the central 50% of the image histogram. In some cases, noise produced by surface reflectance of the specimens or shadows that artificially connected adjacent spots during the binarization of the images were manually eliminated.

In the ImageJ program [35], a macro (Additional file 2) was programmed to automate image processing and measurements. This included 8-bit image conversion, binarization with a minimum automatic threshold, background removal, mask conversion and gap filling. The outlier points, both black and white (using radius 6 and threshold 50) were then removed and the resulting particles (spots) were measured.

Heat maps were obtained by superimposing the images of the spot patterns of all individuals per haplogroup, using the PAT-GEOM v1.0.0 package, developed by Chan et al. [36]. This package allows the analysis of different measures of the coloration pattern quantitatively, and it was designed to work with macros on ImageJ. These maps allowed us to visually explore and qualitatively describe the general patterns that characterized each haplogroup.

### Quantitative characterization of the spot pattern

The spots were automatically numbered consecutively for identification. The central spots were designated spots 1 and 2, respectively, and spots on the edge of the abdomen were numbered consecutively using odd numbers on the left and even numbers on the right. To quantitatively describe the pattern of spots, a series of primary variables were taken at the spot level, as well as derived variables that included both the spot and individual levels.

**Fig. 2.**
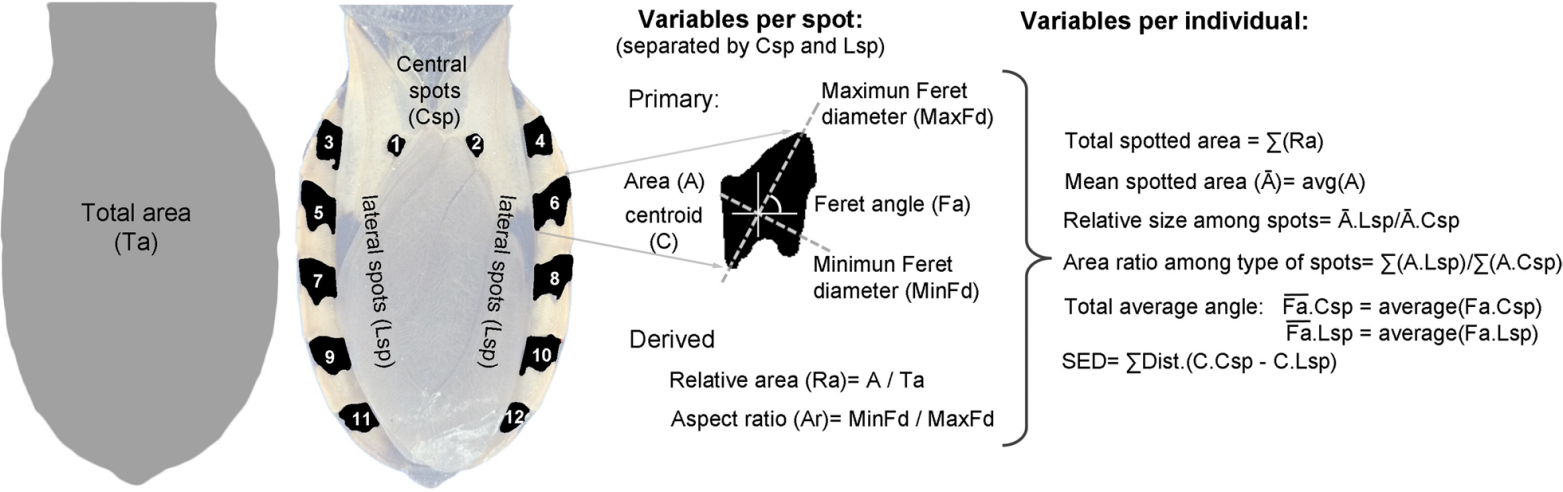
Variables used for the quantitative description of the spot pattern in three haplogroups of *T. dimidiata* (Hemiptera: Reduviidae).

The variables measured are shown in Fig 2. The total body area (Ta) was used for standardization purposes only. The relative area (Ra) is the area of each spot relativized as a percentage of the Ta (%). The sum of the Euclidean distances was calculated by taking the centroid coordinate of each spot and calculating, at the individual level, the distance between the central spots and lateral spots, but only after making a Procrustes record of the complete configurations. The maximum and minimum Feret diameters (MaxFd and MinFd, respectively), as well as the Feret angle, were calculated for each spot. These variables refer to the maximum and minimum distances between any pair of contour points of a shape, and although they are identified as diameter, they are not strictly analogous to a diameter since they do not pass through the center of the figure or divide it into symmetrical sections. The Fa refers to the angle of the vector of the MaxFd and indicates the general directionality of the spot (its inclination). The aspect ratio (Ar) of each spot (ratio of the minor to the major diameter) was used as an indicator of its shape.

For each individual, the averages of the variables per spot, the sum of the total Ra of the spots, and the ratio of the mean Ra of the central spots to that of the lateral spots were calculated as derived variables. For the calculation of the average inclination angle, both for the central spots and lateral spots, the angles of the spots from the left to the right quadrant (0–90°) were recorded.

### Data analysis

Non-parametric descriptive statistics (median, quartiles and range) were used because the distribution of the data was not normal, and traditional descriptors gave a false impression of precision and marked differences. Statistical comparisons among haplogroups were performed using Kruskal–Wallis tests in Statistica v8 software (StatSoft, Tulsa, OK, USA). A linear discriminant function analysis (forward stepwise) (LDFA) was also performed to estimate the ability to discriminate haplogroups based on the variables used. Since this method has a series of restrictive premises and can only linearly differentiate the groups, a multilayer perceptron type neural classification network was used as an alternative method. Neural networks are supervised machine learning procedures and do not have statistical premises on the nature of the data, making them more powerful and capable of exploring nonlinear relationships in complex sets of variables. The option ANS (Automated Network Search) in Statistica v8 software was used to find the topology that most efficiently identified the haplogroups by using all variables. The network was trained with 60% of the individuals by haplogroup and validated with the remaining 40%. Assignment to each group was random, except for individuals wrongly classified by the LDFA, who were forced into the validation sample for a more robust check of network performance. The weight assigned by the neural network to each variable was estimated to identify those of greatest importance in the discrimination process.

## Results

**Fig. 3.**
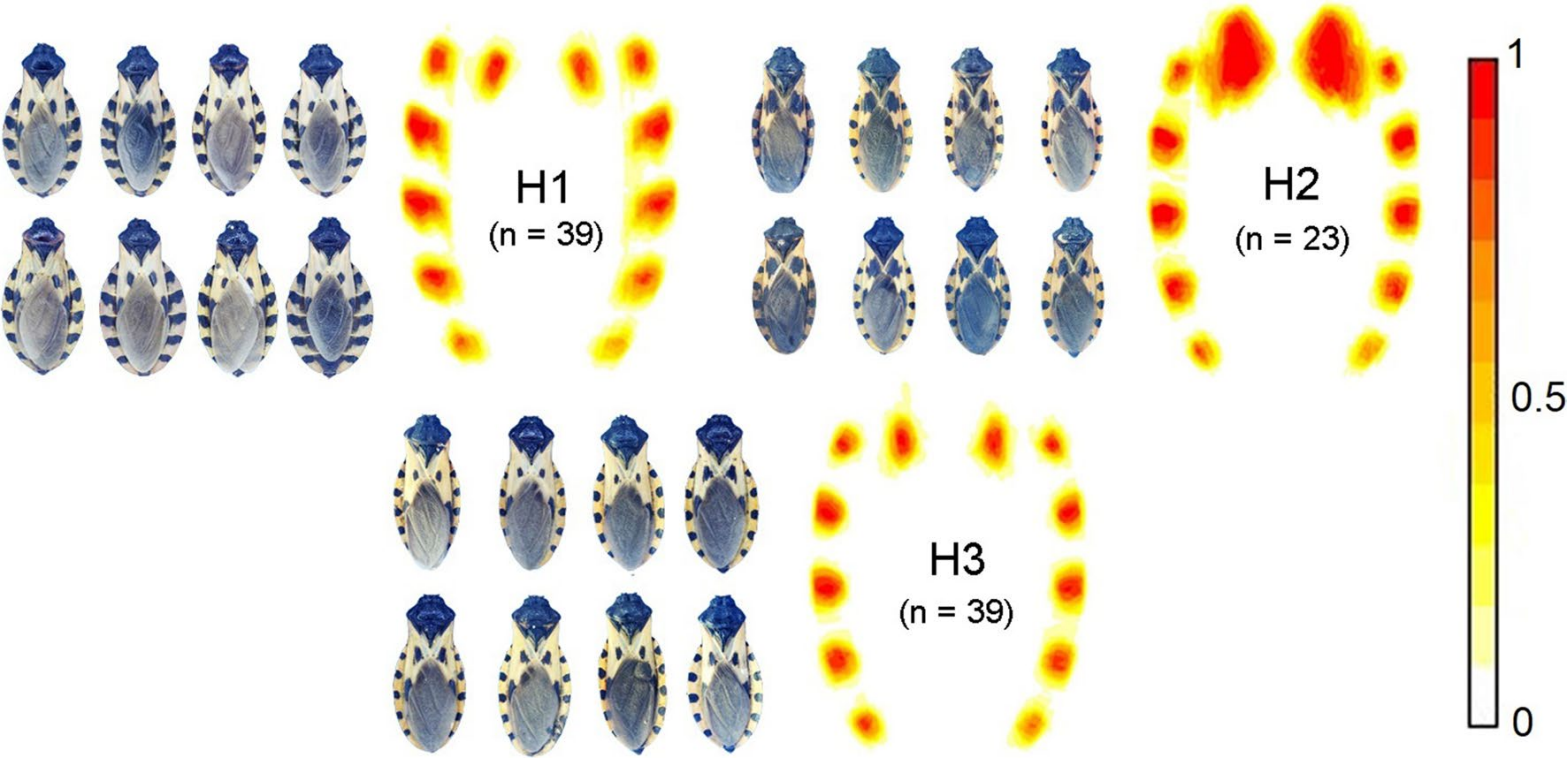
Heat maps obtained by superimposing the spot patterns of all the individuals from the samples of three haplogroups (*H1*,* H2*,* H3*) of *T. dimidiata* (Hemiptera: Reduviidae), and a sample of the images used to visualize the differences between the haplogroups. Heat maps are composed of overlapping individuals by haplogroup. The color scale represents the areas of overlap between spots, with high values representing the most shared pattern among individuals, and low values representing extreme patterns.

The heat maps generated by superimposing all of the individuals within each haplogroup revealed those spot patterns that characterize each haplogroup and provided evidence of a well-differentiated pattern between the haplogroups (Fig. 3). The most differentiated pattern was presented by haplogroup 2, being primarily apparent in the notably larger central spots. Haplogroups 1 and 3 were more similar to each other, but there were consistent differences in the shape and orientation of the spots between these two haplogroups.

**Fig. 4.**
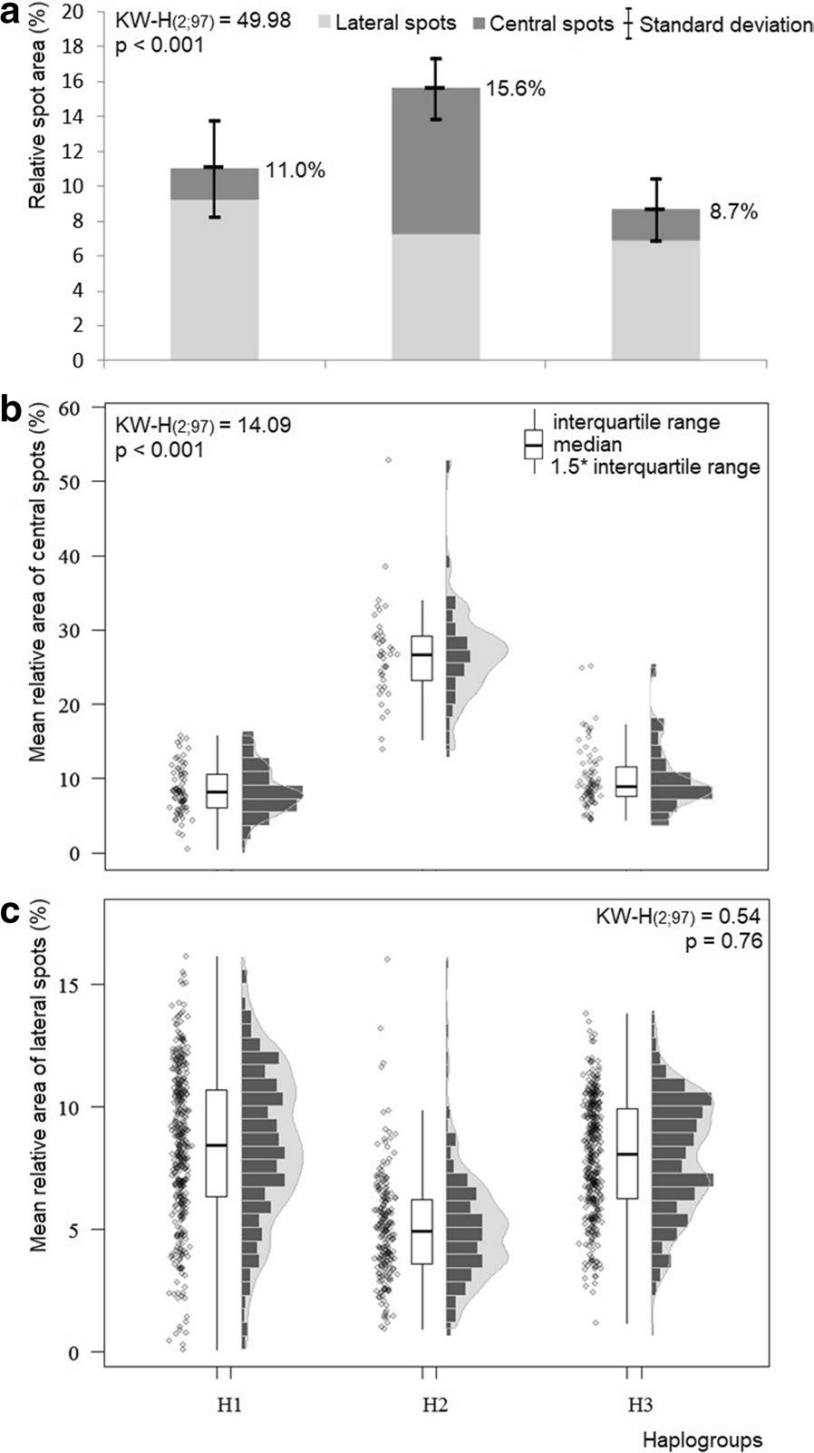
Differences in relative spot area of the abdomen (**a**) and the mean relative areas of the central (**b**) and lateral (**c**) spots in the three haplogroups of *Triatoma dimidiata* (Hemiptera: Reduviidae).* KW-H* Kruskal–Wallis H test

The ratio of spotted area to Ta differed among haplogroups. The highest Ra was presented by haplogroup 2, with 15.6 %, while the Ra for haplogroup 3 was only 8.7 % (Fig. 4a). When comparing the ratio of the area of the central spots to that of the lateral spots, haplogroups 1 and 3 had higher relative lateral spots. In haplogroup 2, the lateral spots and central spots contributed almost equally to the total spot area, while the percentage that the central spot area contributed to total spot area was slightly higher. Statistical comparison of the mean Ra of the central spots and lateral spots revealed that only the central spots differed significantly among haplogroups (Fig. 4b, c).

**Fig. 5.**
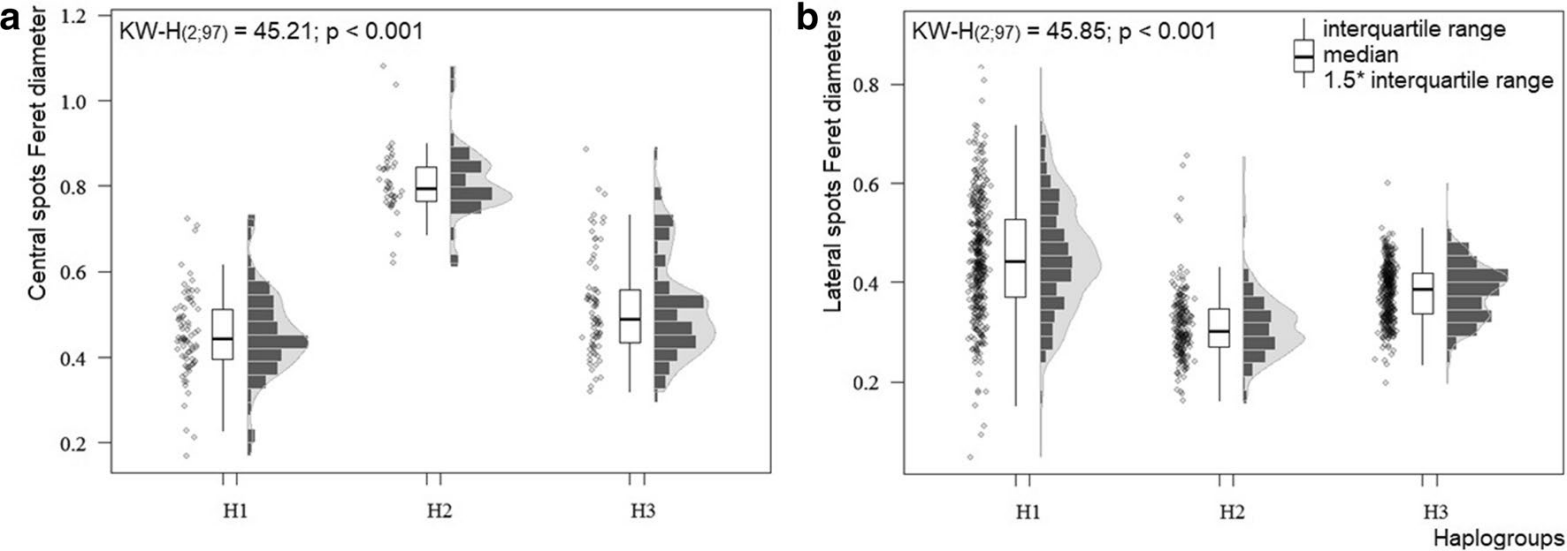
Differences in the average size (Feret diameters), of the central (**a**) and lateral (**b**) spots on the abdomen of three haplogroups of *T. dimidiata* (Hemiptera: Reduviidae)

The average spot size, characterized by Feret diameters, was significantly different among haplogroups, both for the central spots and the lateral spots (Fig. 5). In the case of the central spots, haplogroup 2 was the most strongly differentiated (Fig. 5a), while for the lateral spots, haplogroup 1 presented the most notable differences (Fig. 5b).

**Fig. 6.**
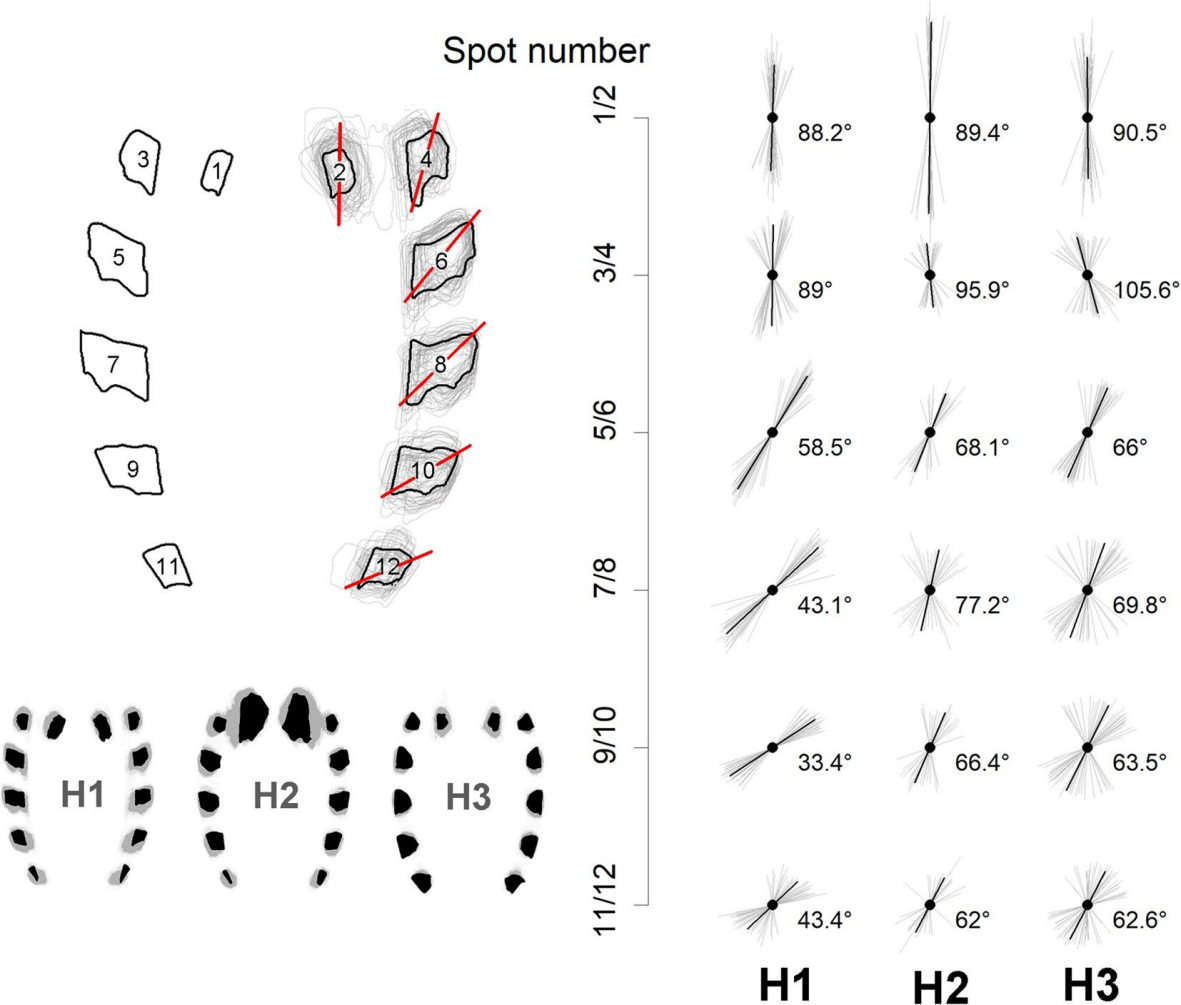
Orientation patterns of the spots (Feret diameter angle). Red and black lines represent the orientation of Feret diameter angle.

The orientation of the abdominal spots, expressed by the Fa, were markedly different between haplogroup 1 and the other groups. The largest differences were observed in the orientations of the first three pairs of lateral spots (3/4, 5/6 and 7/8), which tended to be more forward oriented. For the remaining spots, although differences in orientation were observed, these were less noticeable, both in the Fa value and in its variation among individuals (Fig. 6).

**Fig. 7.**
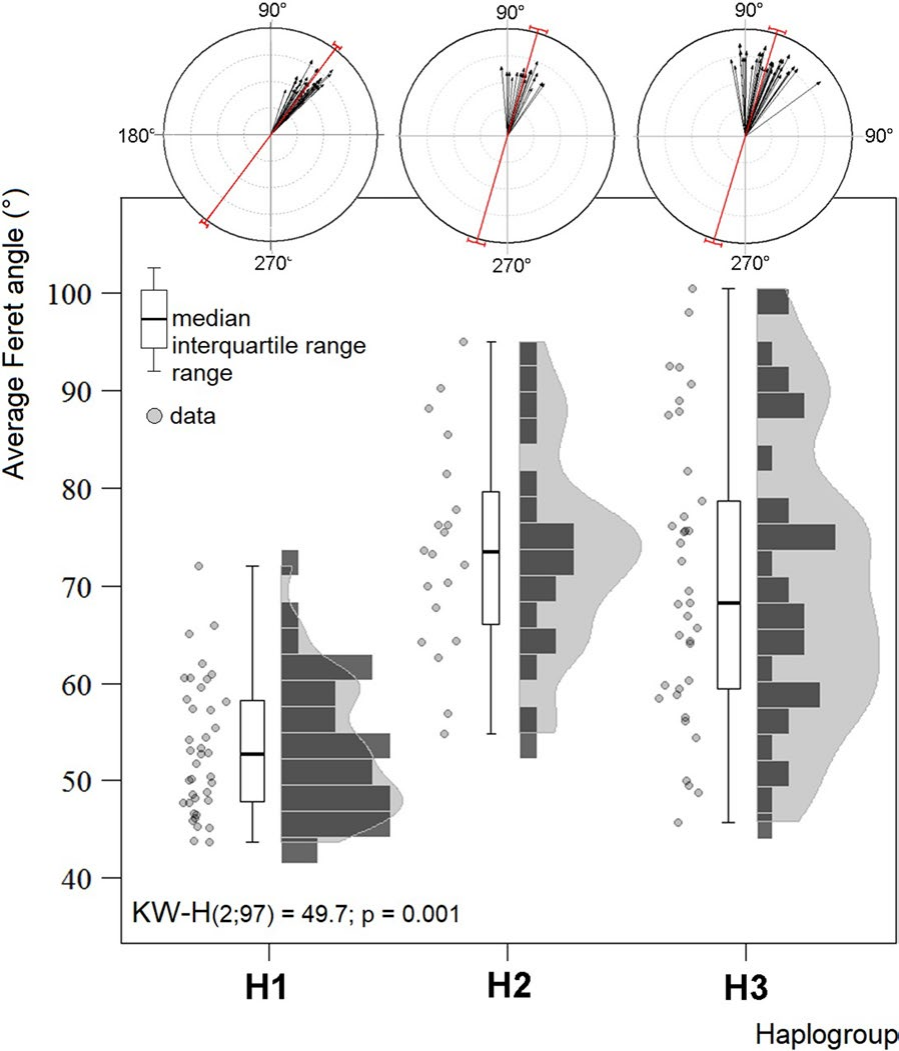
Comparison of the mean orientations of the lateral spots of the abdomen (Feret angle) between three haplogroups of *T. dimidiata* (Hemiptera: Reduviidae)

When comparing the mean orientations of the lateral spots of the abdomen (Fa), significant differences were found between the three haplogroups. Haplogroup 1 was the most distinct and had less variation in Fa than the remaining haplogroups (Fig. 7).

**Fig. 8.**
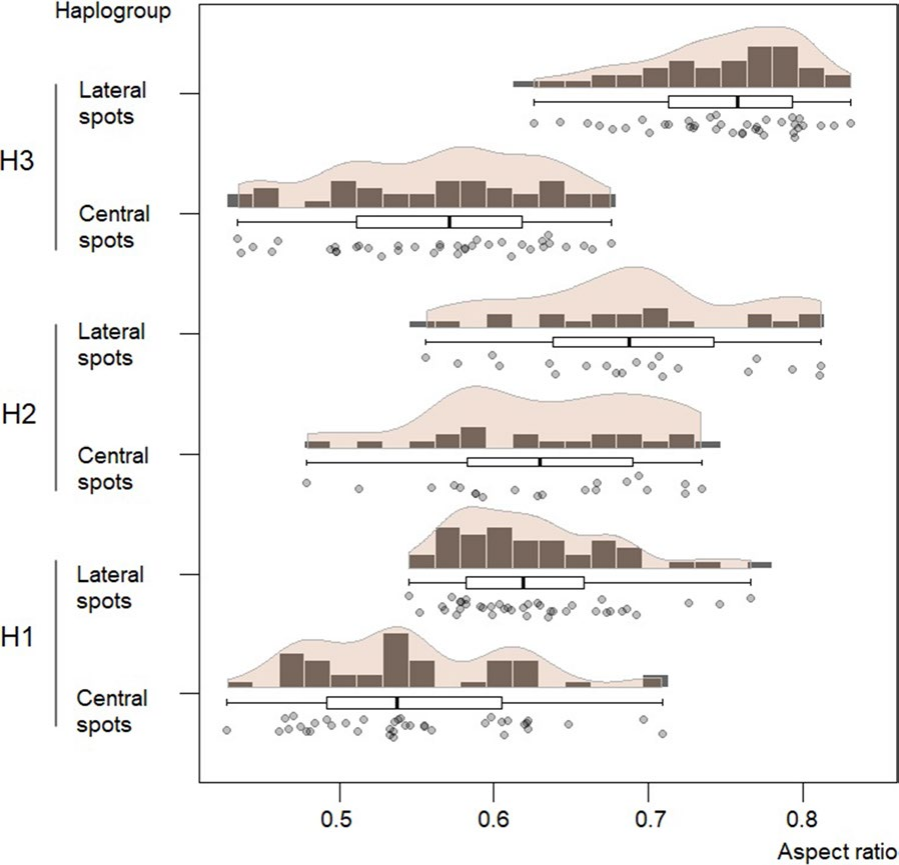
Comparison of the shape (aspect ratio) of the central and lateral spots of the abdomen in the three haplogroups of *T. dimidiata* (Hemiptera: Reduviidae). The aspect ratio differed among haplogroups, both for the central (KW-H_(2; 96)_  = 15.45; *p* < 0.001) and lateral spots (KW-H_(2; 97)_  = 46.25; *p* = 0.001).

The shapes of the central and lateral spots (Ar) differed among haplogroups, both for the lateral and central spots (Fig. 8). The shape of the central spots in haplogroup 2 showed the greatest differences among the haplogroups, while the most differentiated lateral spots were from haplogroup 3.

**Fig. 9.**
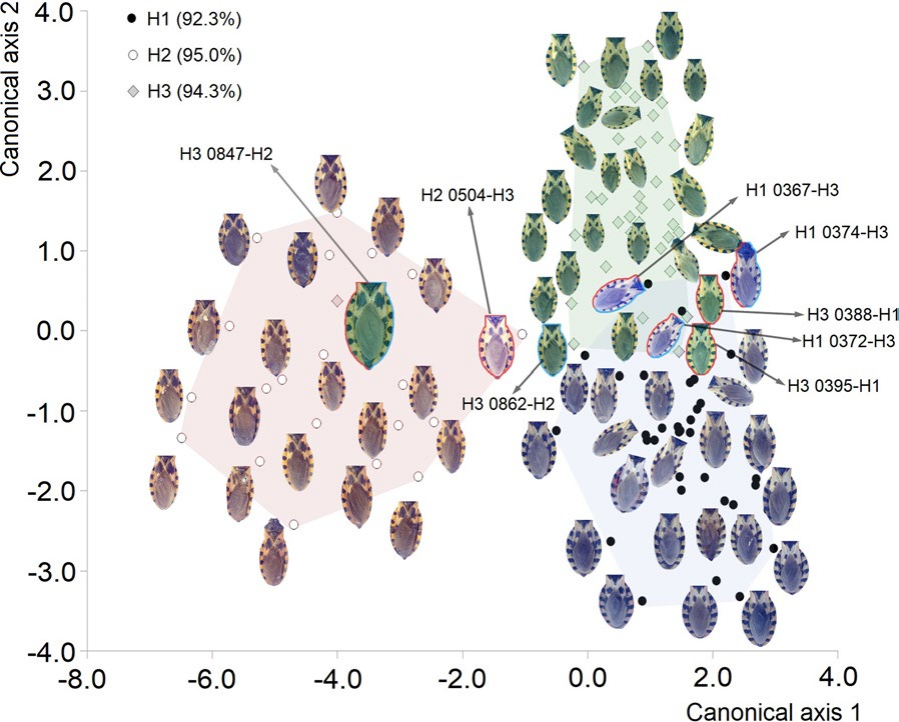
Ordination plot of the discriminant analysis of the individuals of *T. dimidiata* (Hemiptera: Reduviidae) according to the analysis of discriminant function, based on the properties of their spots. The correct classification percentages of the discriminant analysis are shown, and the poorly classified individuals are outlined (red: according to the linear discriminant function analysis, blue: according to the neural network), and their numbers and the haplogroup with which they were confused are given. Full colors represent the minimum convex polygon per haplogroup: blue (H1), pink (H2) and green (H3)

The LDFA correctly assigned 93.8% of the individuals into the correct haplogroups based on spot pattern (Fig. 9). One individual from H2 (H2 0504) was erroneously assigned to H3, and one H3 individual (H3 0847) was misassigned to H2. Haplogroups 1 and 3 had more overlap in the ranking space, with three H1 individuals (H1 0367, H1 0372, H1 0374) assigned to H3 and two H3 individuals (H3 0388 and H3 0395) assigned to H1.

The most efficient neural classification network had a topology with 20 neurons in the hidden layer. This achieved an overall performance of 94.7% with a BFGS-12 training algorithm and an entropy error function. Of the individuals in the training data, 100% were correctly identified; considering only the validation data, 87.2% of the individuals were correctly identified. The hidden layer had sine activation functions and the output layer logistic functions, with sum of squares as an error function. This network achieved 100% correct classification of H2 specimens and misclassified three H1 individuals (H1 0367, H1 0372 and H1 0374; from a total of 16 in the validation sample) as H3, and two H3 individuals (H3 0847 and H3 0862) as H2. The remaining three individuals that had been incorrectly classified by LDFA were correctly assigned to their haplogroups by the neural network (H2 0504; H3 0388 and H3 0395).

Classification methods made similar use of variables. The LDFA used only five variables in the final model: size of the central spots, shape, angle and diameter of the lateral spots and total relative spot area. The neural network assigned greater importance to these same variables and additionally included the relative area of the lateral spots.

**Fig. 10.**
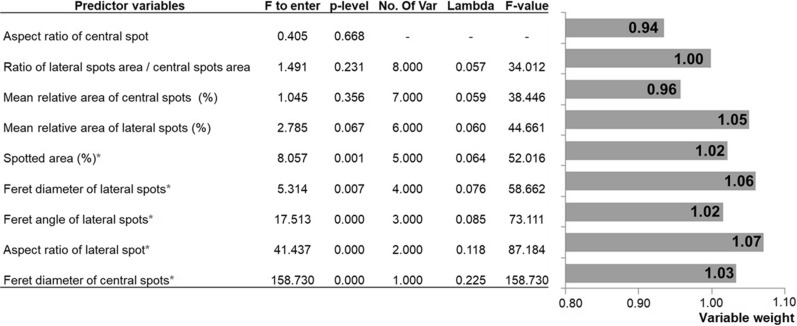
Relative importance of each variable in the classification procedures used to assign individuals to three haplogroups of *T. dimidiata* (Hemiptera: Reduviidae) based on spot pattern. The statistical results of the linear discriminant function analysis are included, in which only the variables identified with a red asterisk and the weights assigned to the variables by the neural classification network obtained were included in the model. Wilks Lambda: 0.057; approximate* F*_(16, 170)_  = 34.01; *p* < 0.001

When analyzing the weights assigned to each variable used in the neural network procedure (Fig. 10), the most important factor in the classification process was the Feret diameter of the lateral spots and the aspect ratio of the lateral spots, respectively. The variable that contributed the least to the classification was the ratio of the central spot area to lateral spot area.

## Discussion

The cryptic *dimidiata* species complex has been largely supported using molecular tools, which has led to the identification of three phylogenetically well-differentiated haplogroups in Mexico and part of Central America [9, 11, 12], and two taxa have been formally described as new species [37, 38]. We report here the first time that the spot pattern presented by this complex has been used to discriminate among haplogroups (possible cryptic species) by extracting and analyzing quantifiable variables from digital images.

Our results demonstrate the ability to use these measures to correctly recognize the haplogroups analyzed. Of the variables used for discrimination, only one (mean relative area of the lateral spots) did not differ significantly among haplogroups, indicating that overall, pattern variables were useful for delimitation. This was verified both by the discriminant analysis ordination plot and the results obtained by the most efficient neural network.

The study of coloration in triatomines and its application in taxonomy has mainly been used in traditional qualitative approaches [32]. This has led to the assumption of a lack of clear morphological diagnostic characters to facilitate recognition and formal descriptions at the species level [15]. However, using heat maps, three well-differentiated spot patterns were evident, corresponding to the three haplogroups. This results once again highlights the importance of using quantitative tools to study complex patterns such as coloration, where subtle aspects, such as the orientation of groups of spots or other patterns, may not be apparent or easily distinguishable to a human observer.

The variation found among the haplogroups in spot pattern may be a response of various different processes. In other groups of insects, such as butterflies, coloration patterns have been shown to vary depending on environmental conditions, such as temperature [39, 40], that are associated with processes of genetic assimilation of phenotypic changes [41]. Although there are populations in which the three *T. dimidiata* haplogroups analyzed in this study are found sympatrically [see 11], their distributions are mostly allopatric; therefore, the pattern of variation among these haplogroups may reflect adaptation to environments with different characteristics in response to environmental stress. Genetic assimilation in the evolution of phenotypic plasticity has recently been demonstrated not only for butterflies but also for various other groups of organisms [42–45]. However, corroborating this phenomenon in *T. dimidiata* will require specifically designed studies.

Another important aspect that this research demonstrates is the value of the combination of digital image analysis and machine learning for taxonomy purposes [46]. The potential of this combination of approaches in species delimitation has been broadly demonstrated [47]. However, even though its utility is clear and, in many cases, superior to the traditional taxonomy, it is still relatively rarely used.

Classical taxonomy is a science that is essentially in danger of extinction, especially due to the lack of expert taxonomists and specialists in species identification, a science which requires many years of training and experience [48]. In the era of big data, image pattern recognition is a new technology that provides many potential advantages for taxonomists, including speeding up and automating the classification process, reducing error and assimilating quantitative information that would be impossible for a human observer [49].

Specifically, with triatomines, there have been recent efforts to employ these methods to establish in automated identification systems. These include the studies of Gurgel-Goncalves et al. [20] and Khalighifar et al. [50], in which geometric morphometry techniques and deep learning algorithms, respectively, were used, representing the first steps toward applying these methods in automated identification systems. Another example is the study of Cruz et al. [26] who were able to discriminate *T. dimidiata* haplogroups with high correct discrimination values by characterizing the entire body contour using Fourier elliptical descriptors; the same method has been successfully used to generate automated identification systems in other groups of insects [51]. The integration of this method with the analysis of the spot pattern is potentially a novel and powerful tool to generate a computer-based approach for species identification in cryptic groups. Although classification processes still need improvement, these novel studies bring new challenges and novel perspectives in the field of epidemiological entomology, and the integration of methods should be a central aspect in the future of automated identification in this group, given its epidemiological importance, as well as in other groups of insects. In the context of haplogroup identification, these methods could generate a whole range of tools that allow their correct identification without the need for genetic testing that is, in most cases, costly and not even possible to perform.

Although the research presented here was focused on evaluating the possibility of correctly discriminating three haplogroups of *T. dimidiata* using the dorsal spot pattern, the value of coloration patterns in species biology cannot be forgotten. Color in insects has important biological functions, including mate choice, intra-sexual competition, dominance relationships and other social interactions [52]. Therefore, the study of color is relevant in many contexts beyond taxonomy, and research should be increased to explore the role that coloration patterns play in nature. In relation to other groups of insects, such as Coleoptera, Lepidoptera and Hymenoptera, in the Hemiptera, and especially the Triatominae subfamily, there are very few studies associated with coloring patterns [52].

## Conclusions

The importance of the correct recognition of insect species of epidemiological importance is vital for the establishment of good control measures [13, 18]. The results obtained in this investigation allow us to conclude that the spot pattern in triatomines constitutes a significant source of information that can be used directly in the taxonomic analysis of this group of insects. If we consider that the haplogroups used here may constitute phylogenetically close cryptic species [9, 11, 53], similar pattern analysis in a larger number of less closely related species will likely find larger, more easily distinguishable differences in the spotting pattern than those found here. A fruitful avenue for future research would be to compare spot patterns among multiple species in order to discriminate among them. If such comparisons are similarly successful as this study, pattern recognition could allow, in the not too distant future, the development of a reliable automated identification system to use as a tool for the recognition of vectors of Chagas disease, one of the most important tropic parasitic disease on the American continents.

## Supplementary Information

**Additional file 1: Figure S1.** Unused individuals in the spot pattern analyses (1.25mb) (https://doi.org/10.6084/m9.figshare.12910007.v1).

**Additional file 2:** Image J macro code programmed to automate image processing and measurements (466b) (https://doi.org/10.6084/m9.figshare.12910010).

## Data Availability

Data supporting the conclusions of this article are included in the article. Also, all data derived from this investigation are deposited in the Figshare repository (https://doi.org/10.6084/m9.figshare.12909968.v1).

## Abbreviations

A: Area
Ar: Aspect ratio
C: Centroid
Csp: Central spots
Fa: Ferret angle
H1, H2, H2: Haplogroups 1, 2, 3
LDFA: Linear discriminant function analysis
Lsp: Lateral spots
MaxFd: Maximun Feret diameter
MinFd: Minimun ferret diameter
SED: Sum of the Euclidean distances
Ra: Relative area
Ta: Total area
