# Supplementary figures and images for: Quantitative imagery analysis of spot patterns for the three-haplogroup classification of Triatoma dimidiata (Latreille, 1811) (Hemiptera: Reduviidae), an important vector of Chagas disease
Source: Parasit Vectors. 2021 Jan 29;14:90. doi: 10.1186/s13071-021-04598-5 (PMC7847135; doi:10.1186/s13071-021-04598-5)

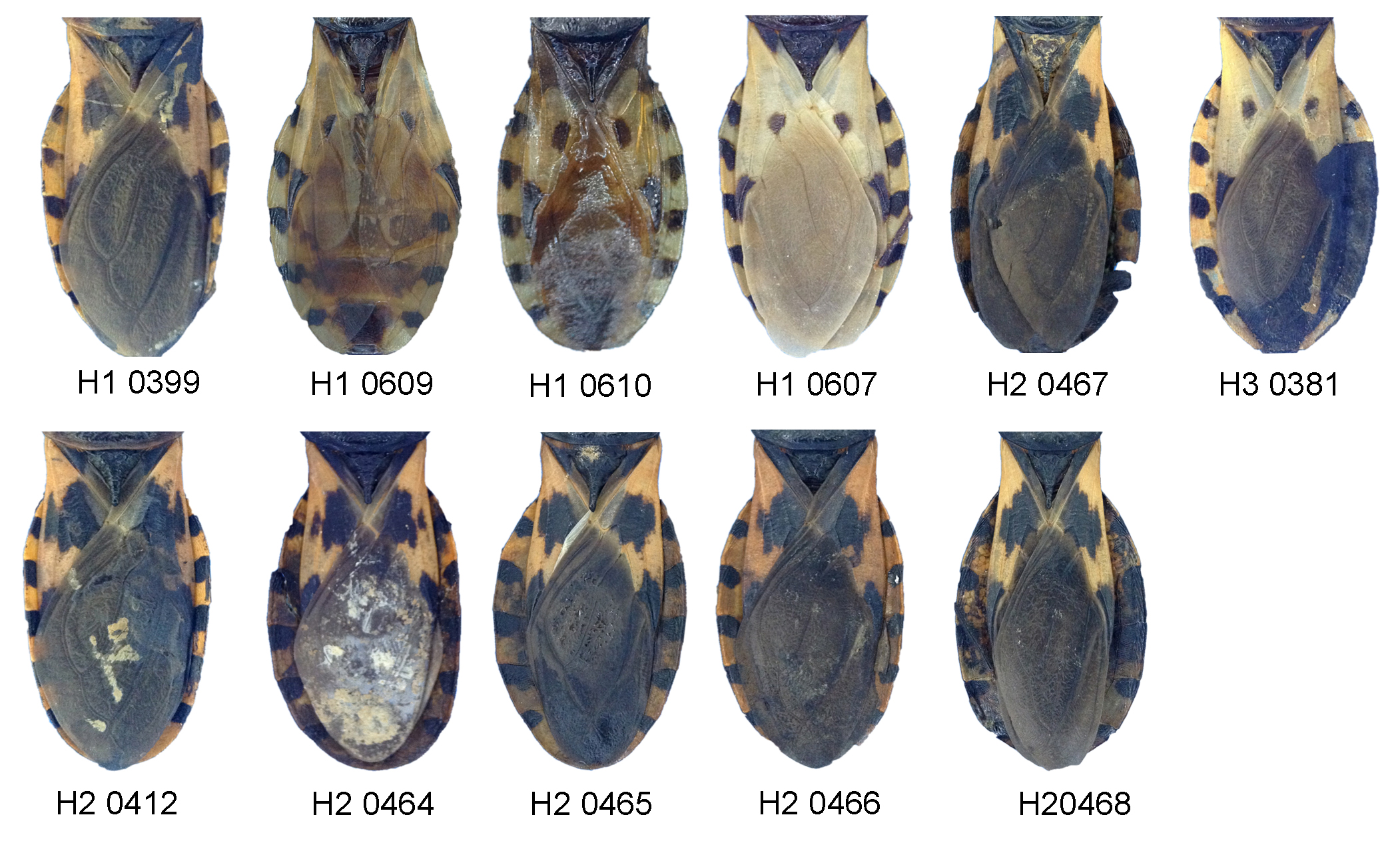

Supplement: Supplementary file 1 — Additional file 1: Figure S1. Unused individuals in the spot pattern analyses (1.25mb) (https://doi.org/10.6084/m9.figshare.12910007.v1). [file 13071_2021_4598_MOESM1_ESM.jpg]
